# Solid Phase Extraction on a Small Scale

**DOI:** 10.6028/jres.093.042

**Published:** 1988-06-01

**Authors:** Gregor A. Junk, John J. Richard

**Affiliations:** Ames Laboratory (USDOE), Iowa State University, Ames, IA 50011

## 1. Introduction

A wide range of sample size, flow rate, cartridge size, and volume of eluting solvent has been reported for the solid phase extraction (SPE) from water of a wide variety of organic compounds. Water sample volumes from 20–2000 mL, flow rates from 2–200 mL/min, cartridge sizes from 100–1200 mg, and volumes of eluting solvents from 0.1–5 mL have been used. At our laboratory, we have been able to achieve a concentration factor of 1000 and recoveries greater than 80% for pesticides [1] and tributyltin chloride [2] present at 0.1 ng/mL in water volumes of 100 mL. These results were obtained using 0.1 mL or less of ethylacetate to elute small cartridges containing 100 mg of C-18 bonded porous silica.

In this paper, we report the verification of this technique by extension to additional pesticides and polycyclic organic materials (POMs) and by field studies of surface and ground water samples where the SPE results are compared to those from conventional, accepted extraction procedures such as Amberlite XAD-2 [3] and solvent extraction.

## 2. Experimental

### 2.1 Extraction Procedures

#### 2.1.1 Pesticides

Cartridges containing 100 mg C-18 bonded porous silica, purchased from J. T. Baker (Phillipsburg, PA), were washed with about 3 mL of ethylacetate, followed by one column volume of methanol which was subsequently displaced with organic-free water. One hundred *µ*L of the standard pesticide spiking solution were added to 100 mL of water which was then forced through the cartridge, using a 50 mL glass syringe, at a flow rate of about 25 mL/min. The C-18 bonded porous silica was then dried by drawing room air through the cartridge using the vacuum from a water aspirator. The adsorbed pesticides were eluted by gravity flow of ethylacetate.

For on-site adsorption of the pesticides from collected surface water samples, the cartridges were preconditioned in the laboratory by washing with ethylacetate and methanol. The methanol was displaced with water and the cartridges were capped and transported to the sampling site where two 50 mL volumes of surface water were passed through each cartridge using a glass syringe. The cartridges were then returned to the laboratory for drying and elution. Duplicate water samples were collected in 4 L amber bottles and returned to the laboratory for processing using the XAD-2 procedure [3].

#### 2.1.2 Polycyclic Aromatic Hydrocarbons (PAHs)

The PAHs were tested at 10 ng/mL using the same procedure used in the pesticide study. Recoveries were checked for partially dried cartridges using 100 μL of benzene eluent. For field samples, solvent extraction with methylene chloride was used for comparison of recoveries with those obtained using C-18 bonded porous silica.

Standard capillary GC analyses were employed for the analyses of all eluates and extracts.

## 3. Results and Discussions

### 3.1 Pesticides

The recovery results from a previous study [1] and new results for some additional herbicides and insecticides are given in table 1.

The combination of the small particle size and high surface area of C-18 bonded porous silica insured contact of the dissolved pesticides with the adsorbent even when very rapid flow rates of up to 250 bed volumes per minute are employed. Our results and those from other investigators [4–8] indicate that flow rate does not have to be closely controlled.

Methanol and acetonitrile have been the recommended solvents for the elution of compounds sorbed to alkyl-bonded porous silicas. Many hydrophobic compounds have limited solubility in these two solvents; consequently 1–5 mL of eluting solvent are usually recommended in the SPE procedures. For pesticides, we found ethylacetate to be a superior eluent capable of recovery >90% of almost all compounds in the first 60 *µ*L of eluate from small cartridges containing 100 mg of 40 μm bonded porous silica.

Comparative field studies are valuable for evaluating the effectiveness of different extraction procedures. In table 2, we report representative SPE results for atrazine, alachlor, metolachlor, cyanazine and metribuzin extracted from 100 mL field water samples taken from four different sites. For comparison, the results are also given for one liter volumes of these same water samples returned to the laboratory for extraction using the XAD-2 procedure [3]. These favorable comparisons for the four sample sites given in the table were also obtained for these same five pesticides in water samples taken from eight other sites located in Iowa and Nebraska.

### 3.2 PAHs

Favorable results, comparable to those discussed above for the pesticides, were obtained in our study of the recovery of PAHs. Good recoveries, as listed in table 3, were obtained when partially air-dried columns were eluted with 100 of benzene.

Preliminary recovery results for other POMs, such as dioxin, trichlorobenzene, fluorenone, anthraquinone, nitrofluorene and nitronaphthalene, showed greater than 90% recovery at 0.1 to 2 ng/mL in water volumes of from 1 to 100 mL.

To further verify the SPE procedure for PAHs, field samples of contaminated water were checked. Comparative values obtained using conventional solvent extraction and SPE are given in table 4. Benzene was used to elute the partially dried cartridges for the SPE procedure and methylene chloride was used for the solvent extraction of the water samples. The excellent agreement of these results is supportive of the more convenient SPE procedure.

## 4. Conclusions

The small size of the C-18 cartridge plus its adaptability to hand-held syringes made it ideal for field sampling. Flow rates did not have to be controlled closely to achieve efficient adsorption even at rapid flows corresponding to 250 bed volumes per minute. Nearly quantitative recoveries were obtained by eluting with as little as 100 μL of organic solvent. In addition to these significant improvements, the SPE procedure as described here, when compared to solvent extraction, retains the following advantages: economical and convenient; uses less organic solvent; requires fewer operational steps; safer because it reduces the use and disposal of toxic and flammable solvents by greater than 90%; amenable to batch processing of samples thus increasing output; and emulsions are not a problem.

## Figures and Tables

**Table 1:** Recovery of pesticides spiked into 100 mL of water

Compounds	%Rec^a^	Compounds	%Rec^a^
Herbicides		Organochlorines	
Alachlor	91	Chlordane	82
Atrazine	80	p p -DDE	81
Cyanazine	92	Endrin	106
Metolachlor	88	Hept. Epoxide	88
Metribuzin	88	Lindane	98
Propachlor	90		
Trifluralin	90	Organophosphorus	
		Chloropyrifos	82
Carbamates^b^		Fonofos	96
Carbaryl	83	Ethyl Parathion	95
Carbofuran	92		

a Avg. Std. Dev. for 4–6 runs was 80%.

b Tested at 1.0 ng/mL; all others at 0.1 ng/mL.

**Table 2:** Results using the C-18 and the XAD-2 method for pesticides in water

Site	Extr Method	mL H_2_O Extr	Concentration in *μ*g/L				
			Atraz	Alach	Metol	Cyana	Metri
1	XAD	3000	23.0	3.0	2.9	3.2	1.3
	C-18	25	22.5	2.9	3.0	3.1	1.1
2	XAD	3000	6.8	1.2	1.6	1.1	0.19
	C-18	25	6.7	1.3	1.2	1.2	0.15
3	XAD	3500	0.31	0.14	ND^a^	0.33	ND
	C-18	100	0.46	0.18	ND	0.42	ND
4	XAD	3500	2.2	0.60	0.37	ND	ND
	C-18	50	1.9	0.81	0.60	ND	ND

a ND = Not Detected.

**Table 3:** Recovery of PAHs spiked into 100 mL of water

Compounds^a^	% Rec^b^
Indene	80
3-Methylindene	82
Naphthalene	83
1-Methylnaphthalene	86
Acenaphthylene	88
Acenaphthene	89
Phenanthrene	97
Fluoranthene	90

a Tested at 2 to 10 ng/mL.

b Avg. Std. Dev. for 4 runs was ±8%.

**Table 4:** Results using C-18 for SPE and methylene chloride for solvent extraction of PAHs

Compounds	Conc in *µg*/L	
	Solv Extn	C-18 SPE
3-Methylindene	13	13
Naphthalene	23	22
1-Methylnaphthalene	436	433
Acenaphthylene	60	69
Acenaphthene	391	376
